# Unmasking Elder Abuse: Depression and Dependency in the Post-Pandemic Era

**DOI:** 10.3390/healthcare12151476

**Published:** 2024-07-25

**Authors:** Isabel Iborra-Marmolejo, Cristina Aded-Aniceto, Carmen Moret-Tatay, Gloria Bernabé-Valero, María José Jorques-Infante, María José Beneyto-Arrojo

**Affiliations:** Meb Lab, Faculty of Psychology, Universidad Católica de Valencia San Vicente Mártir, Avenida de la Ilustración 2, Burjassot, 46100 Valencia, Spain; caded@mail.ucv.es (C.A.-A.); mariacarmen.moret@ucv.es (C.M.-T.); gloria.bernabe@ucv.es (G.B.-V.); mariajose.beneyto@ucv.es (M.J.B.-A.)

**Keywords:** elderly, elderly abuse, depression, dependence, COVID-19, pandemic

## Abstract

The aim of this study was to analyze elder abuse in people over 65 years of age and its relationship with some risk factors—depression symptoms, dependency, gender and age—in the Spanish population. Methods: A battery of questionnaires was administered to a sample of 167 participants electronically (*M* = 72.42; *SD* = 6.46), including the Abbreviated Yesavage Scale to assess depression, the Katz Index for Basic Activities of Daily Living to assess dependency, and the American Medical Association and the Canadian Task Force Questionnaire to assess suspicion of abuse. Results: A prevalence of 40.72% of suspected abuse, of 5.99% of established depression, and of 1.20% of severe dependence was obtained. The prevalence of abuse was higher in the population with dependency (75%) than without dependency (37%). In the case of depression, the prevalence of abuse was 70% for people with established depression and 35.4% for people without depression. Conclusion: Women have higher rates of abuse than men, although this difference is not statistically significant. The same occurs with age. Nevertheless, having established depression and dependency are confirmed risk factors for suffering abuse.

## 1. Introduction

Elder abuse, as defined by the World Health Organization [1] involves any harmful act or lack of appropriate measures within a trusting relationship that causes harm to, or the discomfort of, an elderly person. Elder abuse includes both harmful actions and failures to provide necessary care, occurring in contexts of care, trust, dependency, or co-existence. The perpetrators can be family members, caregivers, friends, neighbors, or institutional staff, with abuse typically occurring in family or residential settings [2].

### 1.1. Prevalence of Elder Abuse

It is difficult to establish the prevalence of elder abuse since it is not as visible as in other age groups. This is mostly due to the less social recognition elder abuse holds, thus handicapping scientific research [3]. The prevalence of elder abuse varies by country. A study carried out in Spain by the Queen Sofía Center [4] stated that 0.8% of the elderly stated that they had been victims of abuse, a figure that reaches 1.5% in the case of dependent elders. Thus, the percentage of abuse increases as the dependence of the elderly increases. At the same time, 4.6% of caregivers of dependent elderly people stated that they had mistreated the person in their charge throughout the year, a figure that increases to 5.7% in the case of elderly people who are highly dependent on their caregivers. Likewise, the numbers of abuse reported by caregivers are much higher than those mentioned by the elderly, except for sexual abuse.

It is also common for elders to suffer more than one type of abuse at the same time [5,6]. According to the victims [4], elders often experience multiple types of abuse simultaneously. According to victims, psychological abuse and neglect are most common, each affecting 0.3%, followed by physical and economic abuse (0.2%), and sexual abuse (0.1%). Dependent elders face higher rates: physical (0.3%), psychological (0.6%), neglect (0.6%), economic (0.9%), and sexual abuse (0.3%). Women are more frequently victims of physical, psychological, and economic abuse than men. Perpetrators report higher rates of economic abuse (1.9%), followed by physical and psychological abuse (1.8%), with increased rates in dependent elders. International studies show psychological and economic abuse at 1.1%, sexual and physical abuse at 1%, and neglect at 0.7%, with an average prevalence of 3%. Cultural stigmatization in Spain may lead to the underreporting of abuse by elders. 

The COVID-19 (coronavirus) pandemic exacerbated these figures, with the overall elder abuse prevalence rising to 21.3%, economic abuse to 7.5%, and physical abuse to 5.4%. A significant portion of victims reported serious incidents and increased frequency of abuse during the pandemic. A study carried out in Poland [7] observed an increase in the rate of abuse of older people during the COVID-19 pandemic, confirming that 45% of the elderly interviewed had been victims of abuse. The types of abuse found were 72.3% psychological abuse, 61.9% neglect, 39.4% of physical abuse, and 36.8% of psychological abuse. Meanwhile, a study has also been carried out in China [8]. A prevalence of 15.4% was observed, which represents an increase compared to levels prior to the onset of the pandemics. Neglect was the most detected type of abuse, followed by economic abuse, psychological abuse, and, lastly, physical abuse. Likewise, 31.2% of the participants stated that they suffered more than one type of abuse.

When evaluating suspicion of abuse, various studies offer different figures in this regard. Prevalences in the Spanish population range between 11.9% [9] and 12.1% [6]. The rates of suspected abuse are higher since they include some situations that may not be exclusive of repeated violence against the elderly.

### 1.2. Risk Factors

According to the literature, the most common risk factors that predispose an older adult to suffering abuse are the following: the victim’s sex and age, the presence of dependency, cognitive impairment and/or psychopathology (and, more specifically, depression) of the elder, the existence of family ties between the victim and the perpetrator, the isolation and lack of social contact of the elder and/or the caregiver, the burnout phenomenon on the caregiver, and the existence of ageism and cultural violence. Moreover, the conditions when victim and perpetrator share a household and/or the perpetrator is economically dependent on the elder are also considered risk factors.

Regarding sex, the risk of being a victim of elder abuse is higher on women [4,6,7,8,9,10]. Women define most of the victims of sexual abuse and the most severe cases of emotional and physical abuse [11]. On the other hand, the study by Risco et al. [10], concludes that the female gender and low autonomy reflected in the Barthel scale are associated with a greater suspicion of abuse. This is a finding that is repeated in the study by Pérez-Rojo [6], where it is noted that the proportion of suspected abuse in women is 15.2%, as opposed to 7.5% in men. The study by Ruiz Sanmartín et al. [9] also states that there is a significant difference in prevalences between men and women. Also, in the case of homicides of older people in which the perpetrator is a relative, female victims are 80% 

The risk of elder abuse increases with age, peaking at 75 years and older [4]. This rise is attributed to the increasing likelihood of developing risk factors with age [11]. Older individuals over eighty, comprising 5.3% of Spanish society in 2014, are at the highest risk [3]. However, some studies suggest that the highest risk is between 60 and 69 years, indicating expert opinion is divided [7,12]. Dependency also heightens the risk of abuse, with higher rates of cognitive or physical deficits among abused elders compared to the general elderly population [11]. Increased dependency correlates with higher abuse rates, particularly economic abuse [4]. There is a synergistic relationship between a victim’s dependency and abuse, where dependency both increases the likelihood of abuse and is a consequence of it [13]. The Reina Sofía Center found that mistreated elders often had disabilities or required assistance more frequently than their non-abused counterparts [4].

Regarding depression symptoms, there seems to be a relationship between depression symptoms and abuse, which is why it is considered a risk factor [7]. It is common to find feelings of unhappiness, shame, guilt, depression symptoms, and suicidal ideation among older victims [11]. At least 10.5% of the elderly had some type of affective disorder [4]. When referring to the perpetrators, they show substance abuse and psychological problems more frequently than non-offending caregivers [11,14,15] and may present a psychiatric history prior to the abuse. The most common disorder in aggressors is depression [11]. Other studies also indicate that a decrease in the physical health of the caregiver and a restriction of his/her freedom of action in his/her daily lives could be related to the appearance of depressive symptoms that would correlate with a greater probability of mistreating the elder due to the appearance of a perceptual bias in the caregiver, who would feel that the elderly person in his/her care is exercising manipulative and controlling behavior towards them [16].

When referring to the victim–perpetrator family ties, research has found contradictory results in this regard. According to a study conducted by the Centro Reina Sofía [4], when the elder is dependent, the abuser is usually one of his/her children, while the elderly person who is not dependent is at a greater risk of being abused by a partner or spouse. Some studies indicate that the perpetrator is usually the elder’s spouse [14], while others confirm that the aggressor is usually a relative of the victim [17]. In the case of sexual abuse, it is very common for the main aggressor to be a family member of the victim or a regular acquaintance, since only 3% of assaults are committed by strangers [15]. Also, the stress suffered by the caregiver when overwhelmed by the situation is a strong predictor of abuse [11,15].

Regarding the financial dependence of the perpetrator and housing conditions, the fact that the offender is economically dependent on the victim is revealed in different investigations [11,15]. Authors consider it the main predictor of abuse in older people, together with the violent behavior of the aggressor that deviates from what is socially correct. In addition, an older person who lives alone presents less risk of suffering abuse than another who lives with a relative.

Isolation and lack of social support are risk factors for both victims and aggressors. These are also important risk factors in the case of sexual abuse [11,15]. has been shown that elders who are victims of violence have fewer social contacts, as do their aggressors, who are said to have difficulties in their social relationships while suffering situations of isolation. In the case of the aggressors, they tend to attribute their social and relationship problems with other individuals to the fact of being a caregiver of an older person. At the same time, aggressors show a lack of social support that causes them to take charge of caregiving by themselves [11].

Ageism refers to negative attitudes and stereotypes towards the elderly that are pervasive in society. These prejudices lead to the dehumanization of older individuals, making them more susceptible to exploitation, mistreatment, and abuse [11]. Giró [3] emphasizes that these societal prejudices contribute to the invisibility of elder abuse. Chang et al. [17] found that 51% of caregivers who mistreated the elderly dehumanized their charges, with a direct correlation between the level of dehumanization and the severity of abuse.

This study aimed to explore the relationship between elder abuse and various risk factors—depression symptoms, dependency, gender, and age—among people over 65 in the Spanish population. Previous research indicates a significant link between depression symptoms and elder abuse, with many victims experiencing negative emotions and mental health issues. Additionally, dependency increases the likelihood of elder abuse, with higher abuse rates among those with cognitive or physical deficits. Financial dependence and living conditions also play a crucial role, with economically dependent offenders more likely to commit abuse. Furthermore, societal isolation and lack of social support are significant risk factors for both victims and perpetrators, especially in cases of sexual abuse. Ageism exacerbates these issues, leading to further invisibility and underreporting of abuse. Thus, in an exploratory way, this study investigated elder abuse within the context of these risk factors to provide a comprehensive understanding of the problem in the Spanish population over 65 years of age.

## 2. Materials and Methods

### 2.1. Participants

To take part in the study, participants should be over 65 years of age. The existence or suspicion of cognitive impairment was considered an exclusion criterion. A sample of 168 participants volunteered to participate in the study, but 1 individual was excluded regarding the inclusion criteria. Thus, the final sample was composed of 167 participants.

In total, 68.6% of the participants were below 75 years of age, while 31.14% were 75 or above. Also, 57.48% were female, while 42.52% were male. When asked about their marital status, 56.28% were married, 14.97% were divorced or separated, 5.38% were single, and 19.76% were a widow/widower, while a 2.99% answered “other”. In addition, 0.59% did not provide any answer.

With regards to educational level, 4.79% had no studies, 13.17% had primary studies, 10.17% had secondary studies, 10.77% had a bachelor’s degree, 11.37% had vocational training, and 49.70% had acquired a university degree. Moreover, 2.99% of the participants were still working, 1.19% were unemployed, 62.27% were retired, and 2.99% answered “other”. In addition, 11.37% of the participants did not provide any answer regarding their job status.

When participants were asked regarding their living conditions, 69.46% claimed to be living with somebody, while 28.74% lived alone, and 1.79% lived in a residence. Out of the 69.46% of elderly people who were accompanied, 0.86% were living with friends; 5.17% were living with their children; 0.86% were living with their children and other relatives; 10.34% were living with their partner and their children; 5.17% were living with other relatives; 74.13% were living with their partner; 0.86% were living with their partner, their children, and other relatives; and 2.58% were living with their partner and other relatives.

In total, 68.86% of the participants did not have antecedents of depression symptoms, while 31.13% did. When individuals were asked about previous infections in relation to COVID-19, 82.63% claimed to have never been infected, while 16.76% had already been ill. In addition, 0.59% refrained from answering.

### 2.2. Measures

To assess depression symptoms among participants, the Abbreviated Yesavage Scale (Martínez de la Iglesia et al., 2002) [18] was employed, which consists of a dichotomous scale of 15 items with an intraobserver reliability of 0.95 and an interobserver reliability of 0.65, and an internal consistency of 0.99.

To assess the level of dependency, the Katz Index (Álvarez et al., 1992) [19] was used. It consists of a dichotomous scale which evaluates six basic activities of the daily life (bathroom, dressing, use of the toilet, mobility, continence, and feeding). It has a reliability of 0.94 and a validity of 0.97.

Finally, to assess abuse, the American Medical Association (AMA) and the Canadian Task Force (CFT) Questionnaire on suspected mistreatment of the elderly was used [9]. This screening instrument consists of nine questions related to different types of elder abuse (physical, psychological, sexual, economical, and neglect of care), with a dichotomous response. One affirmative answer to any of the items is considered indicative of suspected abuse. This questionnaire has a Cronbach’s Alpha of 0.69. In addition, data were collected on the sociodemographic characteristics of the sample (sex, age, and educational level) and on antecedents of depression and social isolation experienced during and after the pandemic (living alone, accompanied, during lockdown, etc.).

### 2.3. Procedure

The current study is an exploratory research project under a cross-sectional design. An incidental snowball procedure was carried out, relying on significant informants. University programs and other associations for older adults collaborated in the recruitment process. The questionnaires were administered in an online format. Informed consent was mandatory. Each participant was guaranteed anonymity and confidentiality of the answers issued with the corresponding processes for the protection of sensitive data.

### 2.4. Design and Data Analysis

Data were analyzed using the software IBM SPSS Statistics for Windows version 23.0 (SPSS, Armonk, NY, USA, 2013). Descriptive statistics were applied onto the distribution of variables presented in absolute and relative frequency tables.

A cluster analysis was performed including z-scores regarding depression symptoms (Yesavage) and age. Abuse was also included as a dichotomous variable. This method enables the generation of data with both continuous and categorical attributes. Cluster analysis was performed under the Log-likelihood test based on Schwarz’s Bayesian inference criterion (BIC). The statistical significance of comparisons was assessed through the Pearson χ^2^ test for proportions. Odds ratios (ORs) with 95% confidence intervals (CIs) were also calculated.

### 2.5. Ethics

This research was approved by the Research Ethics Committee of the Universidad Católica de Valencia San Vicente Mártir (UCV/2020-2021/138).

The participants received an informative form on the goals of the current research. Informed consent was obtained from all subjects involved in the study. The participants were volunteers and were not coerced; they were free to leave at any moment.

## 3. Results

Table 1 depicts sex-differenced rates of abuse (46.88% vs. 32.39%), established depression (7.29% vs. 4.22%), and moderate or severe dependency (7.27% vs. 0%) in the sample of this study. The prevalence of suspected abuse was analyzed, and the results are depicted in Table 2, differentiating between dependent and non-dependent subjects, and subjects with and without depression. The prevalence of suspected abuse is significantly higher in the dependent population, with 75% (9 out of 12 cases) experiencing abuse, compared to 37% (57 out of 155 cases) in the non-dependent population. This indicates a strong correlation between dependency and the likelihood of experiencing abuse.

The results reveal that the prevalence of suspected abuse is highest among individuals with established depression, at 70% (7 out of 10 cases). Those with mild depression have a prevalence of 53.6% (15 out of 28 cases), while individuals without depression show a significantly lower prevalence, i.e., 35.4% (46 out of 130 cases). Other descriptive analyses are provided in Table 2 for the variables under study.

As the variables do not follow a normal distribution, according to the Shapiro–Wilk’s *p*-value, a nonparametric approach through Spearman rho was performed. As Table 3 depicts, a statistically significant correlation between abuse, depression symptoms, and dependency was obtained. The correlation between depression symptoms and abuse was higher (*p* < 0.001) than between abuse and dependency (*p* < 0.01). A statistically significant correlation was also observed between depression symptoms and dependency (*p* < 0.01).

After reviewing the collinearity and random error, a linear regression was performed, as shown on Table 4. The variables sex and history of depression were included as dummy independent variables. In addition, age, depression, and dependency were included as independent variables in the prediction of suspected abuse. The model was statistically significative, indicating that 15% of the variance in suspected abuse can be explained by the included variables: F(5,166) = 5.690; MCE = 5.85; *p* < 0.001; and R^2^ = 0.15.

Finally, the abuse variable was dichotomized between suspicion and absence of suspicion. After a nonparametric approach with the Mann–Whitney U test for independent samples was employed, we examined the relationship between suspected abuse and two factors: depression symptoms and dependency. For depression symptoms, the test statistic (W) is 2166.000, with a *p*-value of less than 0.001, indicating a significant difference. The rank-biserial correlation of −0.357 suggests a moderate negative effect size. For dependency, the test statistic (W) is 3023.500, with a *p*-value of 0.013, also indicating a significant difference, though the rank-biserial correlation of −0.102 suggests a small negative effect size. The regression analysis indicates that both depression symptoms and dependency significantly increase the likelihood of experiencing abuse, with depression symptoms having a stronger effect.

**Figure 1:**
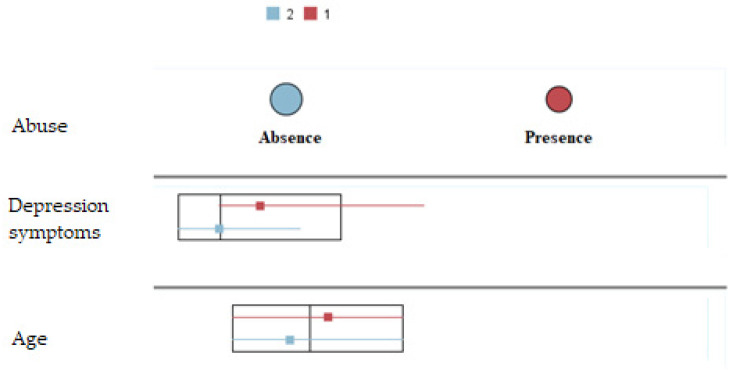
Cluster comparison between the two profiles stipulated. Group 1 (abuse) and group 2 (absence of abuse) across depression and age.

The cluster analysis indicated that a two-cluster solution was the best model, because it minimized the BIC value and the maximum distance between groups. All participants were included and classified in the cluster analysis (100%). Group 1 contained 39.5% of the participants (*n* = 66), while group contained 2 60.5% (*n* = 101). The ratio of sizes was optimal, with a value of 1.53. Figure 1 depicts a cluster comparison for the variables under study. Older participants showed higher scores in depression symptoms and presence of abuse.

Secondly, sex was examined in terms of cluster groups. The Chi-square analysis depicted statistically significant differences between women and men: χ^2^(1) = 5.10; *p* < 0.05. Table 5 depicts a contingency table. The OR of women in cluster number 2 was OR = 2.10; CI 95% [1.09; 4.01]; and *p* < 0.01.

This result suggests that women are more than twice as likely as men to be in cluster number 2, highlighting a significant gender disparity within this cluster.

## 4. Discussion

Considering our main hypotheses, the existence of a positive and significant correlation between suspicion of abuse, depression symptoms, and dependency was confirmed. When it comes to the variables age and sex, a positive but not statistically significant correlation between them and the variable suspicion of abuse was found. Likewise, regarding our hypothesis about history of depression, a correlation was observed, but it lacks sufficient statistical power. In addition, a higher rate of suspicion of abuse was observed among female participants. A higher rate of suspicion of abuse was also found among elderly people with dependency and depression scores.

A prevalence of suspected abuse of 40.72% was observed. Some of the theoretical framework studies offer much lower rates for referring to confirmed situations of abuse [4,8,17,20]. This increase could be because, in the present study, the suspicion of abuse is evaluated and not the abuse by itself, so assumptions that do not constitute abuse in its entirety could have been included. Also, studies that rely on the prevalence of suspected abuse in the Spanish population before pandemics offer prevalences between 11.9% [6,9], so the rate found pre-pandemic is much higher than the prevalence of suspected abuse during the pandemic.

Regarding the prevalence of suspected abuse according to the sex of the victim, women show a higher rate than men (46.88% vs. 32.39%). These scores are consistent with most of the studies reviewed, which showed a higher prevalence of abuse in women [4,6,7,8,9].

Regarding the prevalence of depression, 16.76% of the elderly obtained scores consistent with mild depression, and 5.99% with established depression. This prevalence is somewhat lower than that found in the older pre-pandemic population, which reaches figures of 19.7% from mild depression [21] and 16% from established depression [22]. Regarding the differences between men and women, it was observed that the rates of depression were higher in women than men, obtaining a rate of 20.83% in mild depression and 7.29% in established depression versus 11.27% and 4.22% in the case of men, respectively. 

In addition, a much higher prevalence of suspected abuse was obtained in the case of elderly people with established depression (70% versus 35.4% in the case of elderly people without depression). The elderly with mild depression have a prevalence of abuse of 53.6%. These data are related to what was stated in the previous literature [4,11]. Regarding dependence, its presence was low, with a general prevalence of 2.99% and 1.20% in terms of moderate and severe dependence, respectively. These facts may have influenced the rates obtained, causing them not to be extrapolated to the general population.

It is necessary to mention that all the people who stated that they needed help with one or more of the basic activities of daily living were women, with rates of 5.21% in the case of moderate dependency, and 2.08% in the case of severe dependency. The previous literature offers similar results in this regard, pointing out that women have a higher prevalence than men [22].

A much higher prevalence of suspected abuse on dependent elderly people was also obtained (75% versus 37%) and is also present in the literature [10,11].

On the other hand, the data suggest the existence of a positive correlation between suspicion of abuse and the variables depression and dependency, while the variables sex and age present a positive but not significant correlation; however, older participants showed higher scores in depression and presence of abuse. The influence of depression and dependency on elderly abuse has been previously described in numerous studies [4,8,17,20]. After the first general analysis, no influence of the dependency variable on suspected abuse was observed, but after dichotomizing the suspected abuse variable, differences were found between the subsample with suspected abuse and those without.

Regarding the variables of sex and age, certain age groups are associated with a higher risk of suffering abuse [4,8,17,20]. The literature suggests that being a woman is a risk factor when it comes to being a victim of violence [4,6,8,17,20]. This disparity could be explained by two reasons: most studies do not calculate whether these differences are significant or not, and the fact of being a woman could be a particularly relevant risk factor depending on the type of abuse suffered or its severity. In the case of age, the first explanation could be the cause. According to this, it is crucial to understand every profile separately and the risk associated with each circumstance.

Another variable to take into consideration is the history of depression, which shows a positive correlation, although not significant, with the variable suspicion of abuse. These data reaffirm what was stated in the previous literature [4]. Moreover, validated tools to detect elder abuse in clinical settings are essential for enhancing patient outcomes and ensuring prompt intervention. Cimino and Flanaga [23] emphasize the significance of the Elder Abuse Suspicion Index (EASI) and the Senior AID tool in emergency departments, which enable the rapid and effective identification of abuse in critical-care scenarios. Moreover, investigating the role of technology, such as artificial intelligence (AI) and electronic medical records (EMRs) in improving the identification and documentation of these cases would be advantageous. AI and EMRs can identify injury patterns and automatically alert healthcare providers to potential abuse, marking a substantial advancement in elder care [23]. These technological innovations, when combined with validated screening instruments, can greatly enhance the detection and management of elder abuse, leading to improved protection and care for at-risk elderly populations.

In considering the methodological limitations of the study, it is crucial to acknowledge several key points that may impact the interpretation and generalizability of the findings. Firstly, the reliance on incidental and snowball sampling methods further complicates the generalizability of the findings, as these approaches may introduce biases and limit the representation of the broader elderly population. Additionally, the exclusion criteria targeting severe cognitive impairment and high dependency could skew prevalence rates, preventing the direct extrapolation of results to the entire elderly population. Lastly, the use of self-administered questionnaires can introduce self-report bias, as described in the previous literature [24].

For future research aiming to enhance validity and inclusiveness, it is essential to expand study scopes to encompass individuals without internet access and to encompass all forms of abuse, including sexual abuse, neglect, and violence by strangers. Implementing more inclusive sampling methods that better represent the general elderly population is vital. Furthermore, incorporating individuals with severe cognitive impairment and high dependency, who are particularly susceptible to abuse, would offer a more thorough and precise understanding of abuse prevalence.

The social and clinical implications of these findings are of interest. Clinically, the significant correlation between suspicion of abuse, depression, and dependency underscores the need for comprehensive screening protocols in healthcare settings, particularly for elderly patients. Healthcare providers should be vigilant in identifying signs of abuse, especially in those presenting with depressive symptoms or high dependency levels. Socially, the higher rates of suspected abuse among women and the elderly suggest a need for targeted interventions and support systems tailored to these vulnerable groups. The disparity in abuse prevalence before and during the pandemic highlights the impact of social isolation and increased stressors on abuse rates, suggesting that societal support structures need to be bolstered in times of crisis. Additionally, the findings advocate for more extensive public awareness campaigns to educate communities about the signs of abuse and the importance of supporting those at risk.

In sum, a multidisciplinary approach is essential in managing elder abuse, requiring collaboration among emergency physicians, emergency medical services (EMS), nurses, case management professionals, and primary care physicians for effective identification and treatment. Furthermore, activating community resources and investigative agencies can provide additional support and improve health outcomes for victims. The COVID-19 pandemic has exacerbated the prevalence of elder abuse due to social isolation and increased caregiver stress, underscoring the need for enhanced vigilance and resources to address this critical issue [23]. These improvements would provide a robust and updated foundation for policy recommendations and clinical practices, ensuring a comprehensive and coordinated response to elder abuse.

## 5. Conclusions

The current research supports the primary hypotheses by elucidating a significant interrelationship between suspicion of elder abuse, depression, and dependency within the geriatric demographic. While age and gender demonstrate positive associations with suspicion of abuse, statistical significance remains unattained. Nevertheless, a conspicuous elevation in suspected abuse prevalence among female subjects is discerned, concordant with extant scholarship. The observed prevalence of suspected abuse notably surpasses pre-pandemic metrics, ostensibly attributable to the inclusion of presumptive indicators, thus warranting cautious interpretation. Furthermore, a conspicuous escalation in suspected abuse prevalence emerges among elderly cohorts exhibiting established depression, corroborating the antecedent literature. The dependency incidence in our cohort registers below conventional benchmarks, plausibly owing to our methodological exclusion criteria. Moreover, discernible gender disparities in dependency prevalence corroborate antecedent research. Our findings underscore the imperative of nuanced risk assessment tailored to individual profiles for effective elder abuse mitigation. Additionally, the discerned positive correlation between depression history and suspicion of abuse lends credence to prior scholarly assertions.

## Figures and Tables

**Table 1:** Prevalence of abuse, depression symptoms, and dependency for the participants under study.

Variable	Global	Male	Female
Suspicion of abuse	No suspicion: 59.28% With suspicion: 40.72%	No suspicion: 67.71% With suspicion: 32.39%	No suspicion: 53.12% With suspicion: 46.88%
Depression symptoms	Average: 77.25% Mild: 16.76% Established: 5.99%	Average: 84.51% Mild: 11.27% Established: 4.22%	Average: 71.88% Mild: 20.83% Established:7.29%
Dependency	Absence or mild: 95.81% Moderate: 2.99% Severe: 1.20%	Absence or mild: 100% Moderate: 0% Severe: 0%	Absence or mild: 92.71% Moderate: 5.21% Severe: 2.08%

**Table 2:** Descriptive statistics for abuse, depression symptoms, and dependency.

	Abuse	Depression Symptoms	Dependency
Mean	0.62	3.42	0.15
Std. Deviation	1.08	3.23	0.63
Skewness	3.14	1.18	5.25
Kurtosis	13.22	0.72	30.76
*p*-value of Shapiro–Wilk	<0.001	<0.001	<0.001
Minimum	0.00	0.00	0.00
Maximum	7.00	14.00	5.00

**Table 3:** Spearman’s correlations for abuse, depression symptoms, and dependency.

Variable	Abuse		Depression		Dependency
1. Abuse	—				
2. Depression symptoms	0.35 ***		—		
3. Dependency	0.25 **		0.21 **		—

** *p* < 0.01, and *** *p* < 0.001.

**Table 4:** Coefficients for the regression model in the prediction of suspected abuse. In bold *p*-values lower than 0.05.

Model	Unstandardized	Standard Error	Standardized	t	*p*
(Intercept)	0.65	0.97		0.66	0.50
Depression symptoms	0.07	0.02	0.20	2.59	**0.01**
Dependency	0.27	0.13	0.16	1.98	**0.04**
Sex	0.18	0.16	0.08	1.09	0.27
Depression antecedents	0.35	0.19	0.15	1.85	0.06
Age	−0.01	0.01	−0.04	−0.52	0.59

**Table 5:** Contingency table across sex and cluster groups.

		SEX		Total
		Men	Women	
Cluster	1	21	45	66
	2	50	51	101
Total		71	96	167

## Data Availability

Data are contained within the article.
